# *Jasminum sambac* Cell Extract as Antioxidant Booster against Skin Aging

**DOI:** 10.3390/antiox11122409

**Published:** 2022-12-06

**Authors:** Sara Ceccacci, Adriana De Lucia, Assunta Tortora, Antonio Colantuono, Gennaro Carotenuto, Annalisa Tito, Maria Chiara Monti

**Affiliations:** 1 Department of Pharmacy, University of Salerno, 84084 Fisciano, Italy; 2 PhD Program in Drug Discovery and Development, Department of Pharmacy, University of Salerno, 84084 Fisciano, Italy; 3 Arterra Bioscience SpA, 80142 Naples, Italy

**Keywords:** skin aging, reactive oxygen species (ROS), advanced glycation end products (AGEs), *Jasminum sambac* hydroethanolic extract (JasHEx), mass spectrometry, metabolomics, Global Natural Product Social Molecular Networking (GNPS), nuclear factor erythroid 2-related factor 2 (Nrf2)

## Abstract

Oxidative stress plays a major role in the skin aging process through the reactive oxygen species production and advanced glycation end products (AGEs) formation. Antioxidant ingredients are therefore needed in the skin care market and the use of molecules coming from plant cell cultures provide a unique opportunity. In this paper, the features of an hydroethanolic extract obtained by *Jasminum sambac* cells (JasHEx) were explored. The antioxidant and anti-AGE properties were investigated by a multidisciplinary approach combining mass spectrometric and bio-informatic in vitro and ex vivo experiments. JasHEx contains phenolic acid derivatives, lignans and triterpenes and it was found to reduce cytosolic reactive oxygen species production in keratinocytes exposed to exogenous stress. It also showed the ability to reduce AGE formation and to increase the collagen type I production in extracellular matrix. Data demonstrated that JasHEx antioxidant properties were related to its free radical scavenging and metal chelating activities and to the activation of the Nrf2/ARE pathway. This can well explain JasHEx anti-inflammatory activity related to the decrease in NO levels in LPS-stimulated macrophages. Thus, JasHEx can be considered a powerful antioxidant booster against oxidative stress-induced skin aging.

## 1. Introduction

Genetic intrinsic factors and extrinsic agents such as ultraviolet irradiation, infrared irradiation, xenobiotics and environmental pollutants cause the production of reactive oxygen species (ROS). They are generated by the activation of the mitochondrial respiratory chain, cytochrome p450 and NADPH oxidases [1]. ROS include free radicals (superoxide anion, hydroperoxyl radical, alkoxy radical and hydroxyl radical) and nonradical molecules (hydrogen peroxide, and singlet oxygen) [2]. When antioxidant systems are overwhelmed, ROS accumulate within cells and generate the so-called oxidative stress that is a main contributor to skin aging [3].

Indeed, oxidative stress causes both DNA damage and lipid and protein oxidation together with the triggering of the mitogen-activated protein kinases (MAPK) pathway (AKT, JNK, ERK and p38) [4]. This, in turn, promotes the expression of pro-inflammatory cytokines, growth factors and adhesive molecules through the stimulation of the transcription factors AP-1 and NF-κB. Moreover, MAPK pathway activation causes dermal matrix alterations by reducing collagen levels. In fact, it accelerates collagen breakdown by modulating the expression of matrix metalloproteinases (MMPs) and their tissue inhibitors TIMPs and it reduces the synthesis of new collagen, by blocking the TGF-β type II receptor/Smad signaling. In addition to this, oxidative stress alters skin conditions disrupting the epidermal calcium gradient, essential for the integrity and the function of the cornified envelope, stimulating sebaceous glands function and inducing melanocyte degeneration [5].

In parallel, the formation of modified biomolecules known as advanced glycation end products (AGEs) promotes oxidative stress in cells and tissues [6]. The development of these aging biomarkers is induced by various environmental factors such as cigarette smoke, high levels of refined and simple carbohydrate diets, high-temperature cooked foods and sedentary lifestyle; AGEs are stable and irreversible products derived from the non-enzymatic reaction between reducing sugars and proteins, nucleic acids or lipids, followed by further rearrangements [7]. Indeed, the starting point of the AGE formation is the Maillard reaction in which carbonyl groups of reducing sugars react reversibly with free amino groups of proteins, nucleic acids or aminophospholipids to form Schiff bases. These imines spontaneously rearrange into more stable ketoamines, called Amadori products. They produce reactive dicarbonyls (as glyoxal or methylglyoxal) that react with lysine and arginine functional groups of proteins, yielding a great variety of AGEs [8,9]. They are classified in different groups based on their ability to emit fluorescence and to form crosslinks.

AGE action is mediated by the receptors for AGEs (RAGEs) which are transmembrane proteins belonging to the immunoglobulin superfamily of type I cell surface molecules, expressed in different cell types including keratinocytes, fibroblasts and macrophages. The binding AGEs/RAGEs increases ROS production mainly by the activation of NADPH oxidases (NOXes) and the mitochondrial respiratory chain, leading to oxidative stress and all the above described consequent effects [6,8].

Moreover, extracellular matrix (ECM) proteins, especially collagen, have shown to be highly sensitive to glycation. Indeed, the levels of the AGE structure pentosidine, derived from collagen, are significantly higher in old than in young subjects [10]. Collagen glycation changes not only the mechanical properties of the collagen itself, which becomes stiffer and more brittle [11], but also those of the extracellular matrix, affecting the behavior of the resident cells (growth, differentiation, motility, gene expression and response to cytokines) and matrix–cell interactions [12].

In this scenario, antioxidant ingredients are in great demand in the cosmetic field with the aim of reducing AGE formation and their related oxidative cascade: extracts derived from the specie *Jasminum sambac*, belonging to the Oleaceae family, are interesting for their antioxidant properties and their joined use in folk medicine to treat skin diseases [13]. However, plant extracts display several disadvantages such as the poor bio-sustainability, the potential contamination with pesticides, fertilizers or pathogens and the variability of their qualitative and quantitative composition, affected by seasons and environmental conditions. A valid alternative to plants, as a source of bioactive cosmetic ingredients, is represented by plant cell cultures that, in fact, offer several advantages: (i) high sustainability of the production process, since no agricultural land is needed; (ii) continuous supply of natural products without geographical, season and plant reproductive cycle dependence; (iii) no risks of contamination by pathogens, environmental pollutants and agrochemical residues; (iv) standardized growing conditions that allow to obtain higher and more reproducible rates of biomass and metabolite yield; (v) high versatility, since the concentration of compounds can be optimized by changing culturing conditions and (vi) easier and less time-consuming extraction protocols, reducing the need of aggressive solvents [14,15].

Here, an hydroethanolic extract derived from *Jasminum sambac* cell cultures (JasHEx) was studied. The aim of our study is a broad chemical and biological characterization of JasHEx, particularly exploring its anti-glycation and anti-aging properties. First, advanced mass spectrometric-based approaches were used to obtain a detailed structural characterization of the extract. Then, in vitro and ex vivo experiments were performed to prove JasHEx biological activity as anti-oxidant.

## 2. Materials and Methods

### 2.1. Plant Tissue Cultures and Extract Preparation

A certified Arabian jasmine (*Jasminum sambac*) was obtained from a local nursery (“Ladre di Piante”, Pistoia, Toscana Region, Italy). *Jasminum sambac* leaves were soaked in 70% ethanol (Sigma Aldrich, St. Louis, MO, USA) for 1 min and surface-sterilized with 1% (*v*/*v*) of commercial bleach supplemented with Tween 20 (Sigma Aldrich, St. Louis, MO, USA) for 8 min, followed by three rinses in sterile distilled water. Then, the leaves were excised into 0.5–1.0 cm pieces and cultured on full-strength MS medium [16] containing 3% (*w*/*v*) sucrose, 0.2 mg/L 2,4D and 8 g/L phyto-agar. The explants were monthly subcultured onto a fresh medium for three months. Once a yellow-green, friable and fast-growing callus was obtained, the plant cells were transferred to the liquid MS medium, supplemented with 3% (*w*/*v*) sucrose and 0.2 mg/L 2,4D. The suspension was stirred in a gyratory shaker at 110 rpm and 27 °C in dark climate room. The dark-grown cells were scaled up every week from small-scale to large-scale flasks, until liquid suspension cultures of about 177 g/L were reached. The preparation of *Jasminum sambac* cell culture hydro-ethanolic extract (JasHEx) was carried out by the addition of 2000 mL of a solution ethanol/water (90/10, *v*/*v*) to 500 g of cells. The mixture was homogenized 3 min at 1500 rpm and 6 min at 3800 rpm using a Grindomix GM300 knifemill (Retsch GmbH, Haan, Germany). The obtained suspension was stirred at 400 rpm for 2 h at 25 °C, avoiding light exposure. The suspension was then centrifuged at 6300 rpm for 10 min at 4 °C. The supernatant was removed, filtered and then concentrated under vacuum in a rotary evaporator (IKA RV8, IKA-Werke GmbH & Co., Staufen, Germany) set to 25 °C. Finally, the pH was brought to 7.0 with 10 N NaOH and then freeze-dried until gaining a fine powder.

### 2.2. UPLC–MS/MS Analysis for JasHEx Chemical Characterization

A biphasic butanol/water extraction was achieved. The butanolic fraction was desiccated and thawed in methanol (10 mg/mL) before the UPLC–MS/MS analysis, carried out on a Q-Exactive Classic Mass Spectrometer equipped with an UltiMate™ 3000 UPLC system (Thermo Scientific, Waltham, MA, USA). All the chromatographic runs were carried out as already described by Ceccacci et al. [17].

### 2.3. Global Natural Products Social Molecular Networking Analyses

For metabolite identification, Global Natural Products Social Molecular Networking (GNPS; https://gnps.ucsd.edu; Version 1.3.16-GNPS, UC San Diego on 14 November 2020) was employed [18]. All those MS and MSMS signals not assigned by GNPS were wisely examined and assigned accordingly to literature. Raw files were converted to mzXML format by MS Converter General User Interface software (ProteoWizard; Version 3; http://proteowizard.sourceforge.net/project.shtml; Palo Alto, CA, USA, 94304 on 15 September 2020), before GNPS spectral library search. It was carried out using precursor ion mass tolerance of 0.025 Da, fragment ion mass tolerance of 0.02 Da, minimum matched peaks of 2 and score threshold of 0.7. The results were manually confirmed.

Data pre-processing was carried out by Mzmine (Version 2.53, Softpedia, Bucharest, Romania) [19] and a Feature-Based Molecular Networking (FBMN) job [20] was performed as reported by Ceccacci et al. [17]. The obtained network files were imported into Cytoscape (Version 3.9.1, U.S. National Institute of General Medical Sciences (NIGMS), Bethesda, MD, USA) [21].

### 2.4. Quantitative Analysis of Lignans and Triterpenes

The same UPLC settings stated for the qualitative experiments were employed for the quantitative analysis of lignans, while for that of the triterpenes, they were improved to separate two pairs of isomers, arjunolic/asiatic acid and oleanolic/ursolic acid. The analysis was carried out on a Q-Exactive Classic Mass Spectrometer as previously defined. The separation was carried out by a Phenomenex Kinetex (Torrance, CA, USA) EVO C18 300 Å (150 × 2.1 mm, particle size 5 µm). The mobile phase consisted of A (5 mM ammonium acetate aqueous solution, pH 9.00 adjusted by ammonium hydroxide) and B (100% acetonitrile) using a gradient elution of 13–28% B at 0–20 min, 28–65% B at 20–24 min, 65–75% B at 24–28min, 75–95% at 28–28.5 min, 95% at 28.5–32 min, 95–13% at 32–32.1 min and 13% at 32.1–44 min. The flow rate was 0.450 mL/min and the injection volume was 5 μL. For both lignans and triterpenes, data were acquired with the mass method described by Ceccacci et al. [17]. We purchased nortachelogenin (#LCA52174) from Biosynth Carbosynth, matairesinol (#80497) and maslinic acid (#83209) from PhytoLab GmbH & Co.KG, secoisolariciresinol (#60372) and arjunolic acid (#SMB00119) from Sigma-Aldrich (Saint Louis, MI, USA), asiatic acid (#0027), oleanolic acid (#0041 S) and ursolic acid (#0037 S) from Extrasynthèse (Genay, France). The calibration curves were gained by injecting standards at the concentration of 0.05 to 25 µM for lignans and 0.1 to 25/250 µM for triterpenes. The limit of detection (LOD) and limit of quantification (LOQ) for standards were determined on the basis of the signal to noise (S/N) ratio.

### 2.5. Skin cell Cultures and Explants

Immortalized Human Keratinocytes (HaCaT), bought from Addexbio Technologies (San Diego, CA, USA), were preserved in Dulbecco’s Modified Eagle Medium (DMEM; Sigma Aldrich, St. Louis, MO, USA) that was supplemented with 10% fetal bovine serum (FBS; Sigma Aldrich, St. Louis, MO, USA) in 95% air, 5% CO_2_, and humidified atmosphere at 37 °C. Human dermal fibroblasts (HDF) were preserved in Dulbecco’s Modified Eagle Medium (DMEM; Sigma–Aldrich, St. Louis, MO, USA) supplemented with 10% of fetal bovine serum (FBS; Sigma–Aldrich, St. Louis, MO, USA) in 95% air, 5% CO_2_, and humidified atmosphere at 37 °C. Skin explants, obtained from the skin of healthy female donors (aged 31 and 40) at the surgery center Villa Cinzia (Naples, Italy), were cultured in 24-transwell plates in DMEM/FBS plus antibiotics in air–liquid conditions at 37 °C in 5% CO_2_ humidified air. All donors had given their written informed consent for the use of the skin tissues, according to the Declaration of Helsinki.

### 2.6. Cytosolic ROS Assay in H_2_O_2-_Stressed HaCaT Cells

For this process, 1.8 × 10^4^ HaCaT were seeded in 96-well plates and grown for 20 h. The cells were then treated for 2 h with different concentrations of JasHEx (0.0006%, 0.002% and 0.006% p/v) or with 500 μM ascorbic acid, used as the positive control. After that, they were washed in PBS (Phosphate-buffered saline) and incubated at 37 °C with 100 μL/well of a solution containing: 10 mM of Hepes, 1.3 mM CaCl_2_, 1 mM MgSO_4_, 5 mM of glucose and 5 μM CM-H2DCFDA (5-(and-6)-chloromethyl-2′,7′-dichlorodihydrofluorescein diacetate, Invitrogen). After 45 min, a PBS wash was performed and the baseline fluorescence intensity of the cells was measured at 535 nm (excitation 485 nm), using the instrument EnVision (PerkinElmer, Waltham, MA, USA). Then, the oxidative stress was induced by adding 450 μM H_2_O_2_ and the fluorescence of the samples measured after 30 min.

### 2.7. Enzyme-Linked Immunosorbent Assay (ELISA) for AGE Detection in Glyoxal Stressed Human Dermal Fibroblast (HDF) Cells

For this step, 1.5 × 10^4^ HDF were seeded in 96-well plates and grown for 2 days. After washing with PBS, cells were fixed for 10 min with 100 µL of 4% formaldehyde in PBS. Subsequently, they were treated with JasHEx (0.0006% and 0.002% p/v) or the positive control of 1 µM Aminoguanidine in the presence of 0.5% glyoxal at 50 °C for 1 week. After incubation, they were processed for an enzyme-linked immunosorbent assay (ELISA) using a specific antibody against AGE (abcam ab23722).

### 2.8. ImmunoHistoFluorescence Assay on Methyl-Glyoxal Stressed Skin Explants for Fibrillin-1 Detection

Skin explants were derived from two patients, 31 and 40 years old. From each skin biopsy, three punches were generated for each treatment occurring at the air–liquid interface. The first day, the punches were treated with JasHEx (0.002% and 0.006% p/v) or the positive control (1 mM Aminoguanidine) and after 24 h, 500 µM methyl-glyoxal was added. The treatments were refreshed up each two days to total of seven days. At the end of the period, the punches were processed for histological analysis, fixed in 4% PFA, incubated in 15% sucrose, then in 30% sucrose and cryostored in OCT compound (Optimal cutting temperature) at −80 °C. Cryosection of 5 μm were obtained with the cryostat CM1520 Leica (Leica Biosystems, Buffalo, IL, USA). Slides with cryosections were hydrated for 30 min in PBS and placed in a “blocking” solution (6% BSA, 5% serum, 20 mM MgCl_2_, 0.2% Tween) for 1 h. Subsequently, they were incubated with the primary anti-Fibrillin 1 antibody (MA5-12770, Thermo Scientific, Waltham, MA, USA). for 16 h at 4 °C. The slides were washed with PBS for 30 min and then incubated with the secondary anti-rabbit Alexa-Fluor 546 antibody (A11035, Thermo Scientific, Waltham, MA, USA) for 1 h. The nuclei were stained with DAPI (4′, 6-5 diamidino-2-phenylindole) 1 g/mL in PBS for 10 min. The images were acquired with a fluorescence microscope and analyzed with the ImageJ software (Version 1.53a, National Institutes of Health, USA).

### 2.9. AlphaLISA Assay to Measure Procollagen Type I C-Peptide (PIP) Content

In this step, 8 × 10^3^ HDF were seeded in a 96-well plate and treated for 24 h with JasHEx (0.0006%, 0.002% and 0.006% p/v) or with TGF-β (2.5 ng/mL). After treatment, the cells were processed according to the instructions of alpha LISA hPIP collagen kit provider (AL353HV, (PerkinElmer, Waltham, MA, USA)).

### 2.10. Nrf2 Luciferase-Based Transcription Activation Assay

Here, 6 × 10^3^ HaCaT cells in 96-well plate were seeded and grown for 16 h. After that, they were subjected to a Nrf2 luciferase-based transcription activation assay, using the ARE reporter kit BPS Bioscience (San Diego, CA, USA, #60514). A transfection-ready ARE luciferase reporter vector (containing a firefly luciferase gene under the control of ARE responsive elements located upstream of a minimal promoter) together with an internal control (a constitutively expressing *Renilla* luciferase vector) were transiently co-transfected into HaCaT cells using X-TREME gene HP DNA transfection reagent (Roche, Basilea, Switzerland, #6366244001). After transduction for 24 h, cells were treated for 2 h with the extract (0.0006%, 0.002% and 0.006% p/v) or the positive control Resveratrol (50 μM). After that, they were subjected to the luciferase assay with the Dual-Glo Luciferase Assay System (Promega, Rome, Italy #E2920). Briefly, cells were incubated with firefly luciferase substrate for 10 min prior to measuring luminescence in a 96-well plate reader (Victor Nivo, Waltham, MA, USA). The ratio of luminescence from firefly and Renilla was calculated to normalize and compare Nrf2 transcriptional activity.

### 2.11. Analysis of the Expression of SOD-1(NM_000454.5) and OH-1(NM_002133.3) Genes in HaCaT Cells

For this process, 1.5 × 10^5^ HaCaT cells per well were grown in 6-well plates for 16 h and incubated for 6 h with the extract (0.002% and 0.006% p/v) or 50 μM Resveratrol as the positive control. At the end of incubation, total RNA was extracted using the “GenElute™ Total RNA Purification” kit (from Sigma-Aldrich (Saint Louis, MI, USA) and treated with DNase I (Thermo Scientific, Waltham, MA, USA) at 37 °C for 30 min, to remove genomic DNA contaminant. 500 ng of total RNA was retro-transcribed using the enzyme Reverse transcriptase (Thermo Scientific, Waltham, MA, USA). Semi-quantitative RT-PCRs were conducted using the pair of universal primers 18S primer/competimer (Invitrogen- Thermo Scientific, Waltham, MA, USA) as internal standards. The PCR products were separated on 1.5% agarose gel, viewed using the iBright instrument (Invitrogen- Thermo Scientific, Waltham, MA, USA). The sequences of the primers used for amplification were the following: HsSOD1Fw: GAAAGTAATGGACCAGTGAAGG; HsSOD1Rv: ATTGGGCGATCCCAATTACACC; OH-1Fw GAACTTTCAGAAGGGTCAGG; OH-1Rv GCTCAATGTTGAGCAGGAA.

### 2.12. Nitric Oxide Assay in LPS-Stimulated RAW 264.7

NO concentration was determined in RAW 264.7 murine macrophages, seeded at a concentration of 1.5 × 10^5^ cells/well in 96-well plates for 24 h, and pre-treated with the extract (0.0006%, 0.002% and 0.006% p/v) or with 10 μM TPCK (positive control) for 2 h, before the incubation with 2 μg/mL LPS for 18 h. The amount of NO, converted into nitrite, was calculated by adding Griess reagent (solution of N-(1-naphthyl)ethylenediamine and sulfanilic acid, Invitrogen- Thermo Scientific, Waltham, MA, USA) and, after 30 min, the absorbance was measured at 540 nm by the multiwell-plate reader (EnVision, PerkinElmer, Waltham, MA, USA).

## 3. Results

### 3.1. Qualitative and Quantitative Analysis of Jasminum sambac Cell Culture Hydro-Ethanolic Extract (JasHEx)

**Figure 1:**
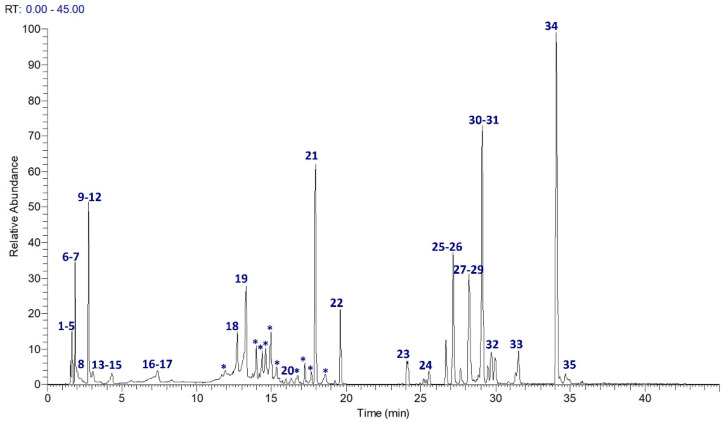
Extracted ion chromatogram of the main metabolites identified in JasHEx. Those indicated by * were included in Molecular Networks (MNs).

**Figure 2:**
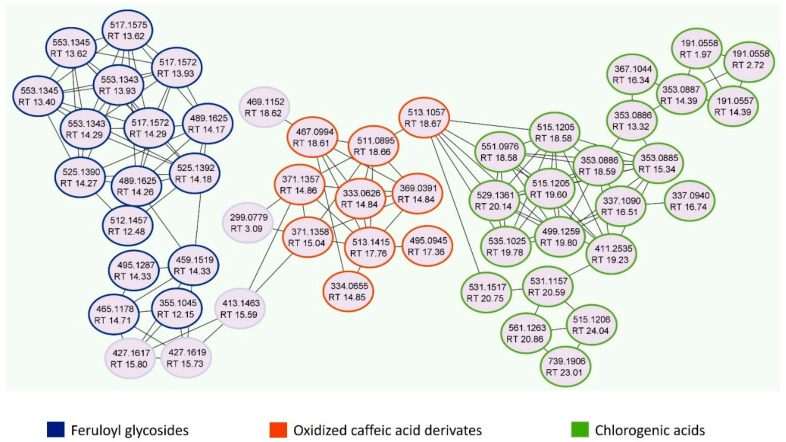
Molecular networks (MNs) showing the presence of chlorogenic acids, oxidized caffeic acid derivates and feruloyl glycosides in JasHEx.

UPLC–MS/MS analysis of JasHEx was performed and high-resolution spectrometric data were analyzed using Global Natural Products Social Molecular Networking (GNPS), a web-based mass spectrometry system that aids in the annotation of natural products (NPs) [18]. In particular, a GNPS spectral library was performed to achieve online dereplication. Chemical species not identified by GNPS were assigned accordingly to the literature. As shown in Figure 1 and Figure 2 and Table 1, more than 50 compounds belonging to several classes of secondary metabolites, mainly polyphenols and terpenes, were identified. Indeed, JasHEx extract contains phenolic acid derivatives, lignans (secoisolariciresinol, nortrachelogenin and matairesinol) and triterpenoids (arjunolic acid, asiatic acid, maslinic acid, oleanolic acid and ursolic acid).

Furthermore, a Feature-Based Molecular Networking (FBMN) job was also carried out. It is able to group related NPs within a network since structurally similar molecules share similar MS/MS fragmentation patterns [20]. The FBMN job allowed us to identify several chlorogenic acids, reported in Figure 2 and Table S1 and circled in green. Compounds eluted at 13.32, 14.39 and 15.34 min and generating the same deprotonated ion [M-H]^−^ at *m*/*z* 353.09 were recognized as mono caffeoylquinic acids (CQA, C_16_H_18_O_9_, mass error of 5.38 ppm). Indeed, MS^2^ ions at *m*/*z* 191.06 and 173.04 correspond to deprotonated and dehydrated quinic acid, while fragments at *m*/*z* 179.03 and 135.04 derive from the deprotonation and decarboxylation of the caffeoyl moiety. The MS^2^ base peak of the species with RT 15.34 min at *m*/*z* 173.04 allowed its assignation as 4-CQA. The other two compounds provided the same MS^2^ base peak ion at *m*/*z* 191.06, but the intensity of the fragment ion at *m*/*z* 179.03 allowed to identify the first compound (RT 13.32; *m*/*z* 179.03 intensity of 40%) as 3-CQA and the second (RT 14.39; *m*/*z* 179.03 intensity of 4%) as 5-CQA [23].

The compound with RT 16.34 min and parent ion at *m*/*z* 367.10 (C_17_H_20_O_9_, mass error of 5.45 ppm) is a feruloylquinic acid (FQA): MS^2^ ions at *m*/*z* 193.05 and 134.04 derive from the deprotonation and from demethylation plus decarboxylation of the feruloyl moiety, respectively. The MS^2^ base peak at *m*/*z* 191.06, corresponding to deprotonated quinic acid, allowed its assignation as 5- FQA [23].

Species eluted at 18.58 and 19.60 min and generating the deprotonated ions [M-H]^−^ at *m*/*z* 515.12 are dicaffeoylquinic acids (di-CQA, C_25_H_24_O_12_, mass error of 4.08 ppm). They shared the same MS^2^ base peak at *m*/*z* 173.04 that suggests a substitution at position 4. The second compound (RT 19.60 min) was identified as 3,4-diCQA for the presence of the fragment ion at *m*/*z* 335.08 [CQA-H_2_O-H^+^]^−^, absent in the MS^2^ spectrum of the first one (RT 18.58 min), assigned to 4,5-diCQA [23].

The compound with RT 16.74 min and parent ion at *m*/*z* 337.09 was identified as 4-coumaroyl-quinic acid (4-pCoQA, C_16_H_18_O_8_, mass error of 6.53 ppm), as suggested by the MS^2^ base peak at *m*/*z* 173.04. Ions at *m*/*z* 499.13 and 529.14 eluted at 19.80 and 20.14 min were assigned to 3-coumaroyl-4-caffeoylquinic acid (C_25_H_24_O_11_, mass error of 4.81 ppm) and a 4-caffeoyl 3-feruloylquinic acid (C_26_H_26_O_12_, mass error of 3.78 ppm). The fragments at *m*/*z* 173.04 (base peak) and at *m*/*z* 353.09 suggest a caffeoyl moiety at position 4, whereas those at *m*/*z* 119.05 and 134.04 indicate the presence of a coumaroyl and a feruloyl moiety, respectively, at position 3 [23].

The ions shown in Figure 2 and Table S1 and circled in orange were assigned to oxidation products of caffeic acid derivates, since they shared the same MS^2^ peaks at *m*/*z* 177.02 and 133.03. In particular, species with parent ion at *m*/*z* 513.11 (RT 18.67 min) and 511.09 (RT 18.66 min), showing the MS^2^ peak at *m*/*z* 351.07, were identified as mono (C_25_H_22_O_12,_ mass error of 5.65 ppm) and di-oxidized forms of di-CGA (C_25_H_20_O_12,_ mass error of 4.70 ppm).

The ions at *m*/*z* 333.06 (RT 14.84 min) and m/z 495.09 (RT 17.36 min), generating the MS^2^ fragment at *m*/*z* 93.03, were assigned as mono-oxidized forms of caffeoyl quinic acid lactone (or caffeoyl skimic acid, C_16_H_14_O_8_, mass error of 6.31 ppm) and dicaffeoyl quinic acid lactone (or dicaffeoyl skimic acid, C_25_H_20_O_11_, mass error of 4.65 ppm), with which they coelute [22].

Moreover, the extract contains feruloyl glycosides, circled in blue in Figure 2 (Table S1). In the MS2 spectrum they showed fragments at *m*/*z* 193.05 and 175.04, characteristic of the feruloyl moiety and/or at *m*/*z* 337.09, due to the loss of hexosyl moiety and one molecule of water and/or at *m*/*z* 295.08, 265.07 and 235.06, derived from by cross-ring cleavage of the remaining sugar residue [24]. In particular, the species at *m*/*z* 517.16 (RT 13.62, 13.93 and 14.29 min) were feruloyl disaccharides (C_22_H_30_O_14_, mass error of 3.87 ppm) and those at *m*/*z* 355.10 (RT 12.15 min) were feruloyl monosaccharides (C_16_H_20_O_9_, mass error of 5.91 ppm). The ions at *m*/*z* 489.16 (RT 14.17 and 14.26 min) and *m*/*z* 459.15 (RT 14.33 min) could be ferulic acids combined with a hexose and a C5 polyalcohol (C_21_H_30_O_13_, mass error of 4.50 ppm) or a C4 polyalcohol (C_20_H_28_O_12_, mass error of 4.79 ppm), respectively.

Finally, the identified lignans and triterpenes were quantified. Quantification methods were validated as reported in Table 2. Nortrachelogenin and ursolic acid are the most abundant lignan and triterpene, respectively, present in JasHEx (Table 3).

### 3.2. Cytosolic ROS Detection in H_2_O_2-_Stressed HaCaT Cells

**Figure 3:**
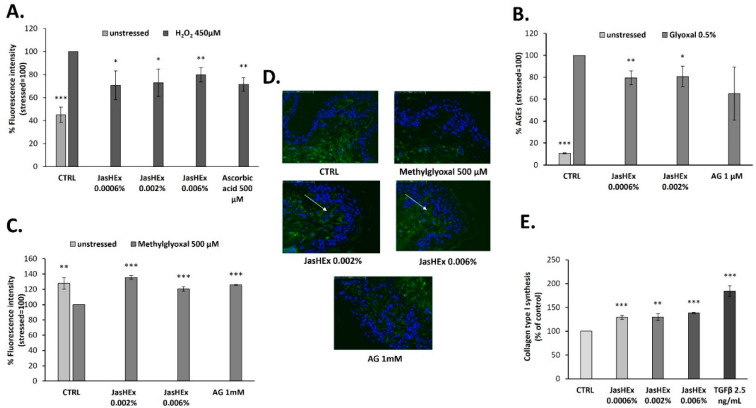
Bar graphs showing JasHEx ability (**A**) to decrease cytosolic ROS levels in H_2_O_2_-stressed HaCaT cells, (**B**) to inhibit AGE formation in glyoxal treated HDF and (**C**) to increment fibrillin-1 content in methylglyoxal stressed skin explants. Panel C has been obtained measuring the fluorescence intensity related to the photographs of skin sections (**D**) in which fibrillin-1 has been highlighted with a specific antibody labeled with fluorophore (green) and the nuclei (blue) have been stained with 4′,6-diamidine-2-phenylindole (Dapi). Panel (**E**) shows the increase of collagen type I synthesis. The bars represent the standard deviations and the asterisks indicate significant variations according to Student’s t-test (* *p* < 0.05, ** *p* < 0.01, *** *p* < 0.001).

Since the large majority of secondary metabolites identified in the extract such as chlorogenic acids, lignans and triterpenes possess well-known antioxidant activity [25,26,27], the JasHEx effect on cytosolic ROS was evaluated. Therefore, HaCaT cells were treated for 2 h with the extract (0.0006%, 0.002% and 0.006% p/v) or with the positive control ascorbic acid (500 µM), incubated with an indicator for ROS and then stressed with H_2_O_2_ (450 μM). After oxidation, the indicator yields a fluorescent adduct. As shown in Figure 3A, the H_2_O_2_-induced stress increased cytosolic ROS formation by 50% and this was reduced by almost 30% both in case of ascorbic acid and JasHEx pretreatment.

### 3.3. AGE Detection in Glyoxal Treated HDF

Since AGE formation is dependent on oxidation reactions, JasHEx anti-glycation activity by an enzyme-linked immunosorbent assay (ELISA) was evaluated. It allowed us to detect, by a specific antibody, AGE products in human dermal fibroblasts (HDF), treated or not with the extract (0.0006% and 0.002% p/v), in the presence of 0.5% glyoxal at 50 °C for one week. Aminoguanidine (AG) 1 µM was used as the positive control. The treatment with 0.5% glyoxal stimulated the formation of AGE products by 90%, whereas the incubation with JasHEx at both concentrations was able to reduce it by 20% (Figure 3B).

### 3.4. Fibrillin-1 Detection in Methylglyoxal Stressed Skin Explants

On the basis of these results, to confirm the antiglycation effect in a physiological context, the JasHEx effect was tested on methylglyoxal stressed skin explants. Since fibrillin-1, an ECM protein essential for the dermal elastic network, is highly sensitive to glycation, we decided to use it as a biomarker. Indeed, the increase in fibrillin-1 glycation induced by methylglyoxal significantly alters its conformational structure and the modified protein is no longer recognized by the used antibody [28]. Therefore, skin explants were treated with JasHEx (0.002% and 0.006% p/v) before and after 500 µM methylglyoxal addition and the content of fibrillin-1 was detected by Immuno-Histo-Fluorescence assay. Aminoguanidine (AG) 1 mM was used as the positive control. As shown in Figure 3C and D, the addition of 500 µM methylglyoxal reduced fibrillin-1 levels by 30%. The incubation with the extract was able to protect fibrillin-1 from methylglyoxal- induced glycation. In particular, the treatment with JasHEx at the concentration of 0.002% p/v increased fibrillin-1 content by 35%, similar to the positive control.

### 3.5. Analysis of Collagen Type I Synthesis

To measure the JasHEx effect on the synthesis of collagen type I, further than its protective effect from glycation, Procollagen Type I C-peptide (PIP) was used as indicator. Indeed, the collagen type I is synthesized as procollagen that contains peptide sequences (propeptides) at both the amino-terminal and carboxy-terminal ends, essential for the winding of procollagen into triple helix. These propeptides are cleaved during secretion and the triple helix collagens polymerize into extracellular fibrils [29]. Therefore, the amount of released PIP stoichiometrically reflects the amount of synthesized collagen. Thus, HDF were treated for 24 h with JasHEx (0.0006%, 0.002% and 0.006% p/v) or with TGF-β (2.5 ng/mL), used as the positive control, and processed for an AlphaLISA assay to measure PIP levels. As shown in Figure 3E, the incubation with all extract concentrations significantly increased the content of PIP.

### 3.6. Analysis of Nrf2/ARE Pathway in HaCaT Cells

**Figure 4:**
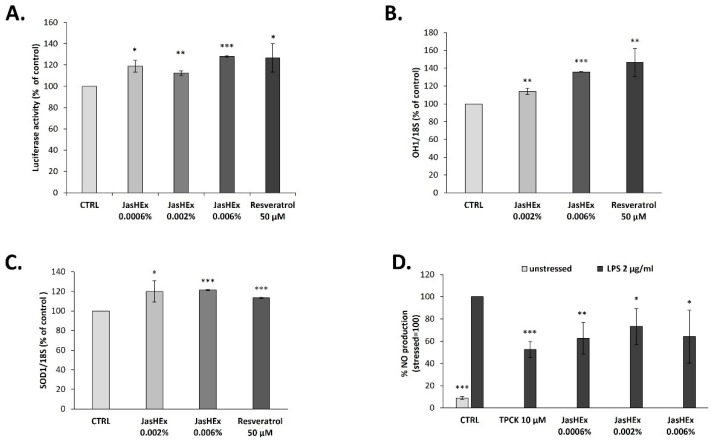
Bar graphs showing JasHEx effect (**A**) to activate Nrf2/ARE signaling, (**B**) to increase OH-1 and (**C**) SOD-1 gene expression in HaCaT cells and (**D**) to reduce NO production in LPS-stimulated RAW 264.7 cells. The bars represent the standard deviations and the asterisks indicate significant variations according to Student’s t-test (* *p* < 0.05, ** *p* < 0.01, *** *p* < 0.001).

Due to the antioxidant and antiglycation activity of JasHEx, the effect of the extract on Nrf2/ARE (nuclear-related factor 2/antioxidant response element) pathway was investigated, since it is the most pivotal endogenous antioxidative system studied so far [30]. Therefore, an Nrf2 luciferase-based transcription activation assay on HaCaT cells, incubated for 2 h with the extract (0.0006%, 0.002% and 0.006% p/v) after transduction, was performed. The treatment with 0.006% p/v JasHEx increased luciferase activity linked to Nrf2 by 28% (Figure 4A), similar to 50 µM resveratrol, used as the positive control.

### 3.7. Analysis of OH-1 and SOD-1 Gene Expression in HaCaT Cells

The effect of JasHEx in HaCaT cells on the expression of Nrf2 gene targets such as Superoxide dismutase 1 (SOD-1) and Heme oxygenase-1 (HO-1) was also tested. To do this, HaCaT cells were treated with JasHEx (0.002% and 0.006% p/v) for 6 h and then SOD-1 and OH-1 expression was analyzed by RT-PCR. The results, reported in Figure 4B,C, demonstrated that the extract, increased the expression of both genes, as the positive control resveratrol (50 µM).

### 3.8. NO Determination in LPS-Stimulated RAW 264.7 Cells

Since Nrf2 also plays a role in counteracting NF-κB-driven inflammatory response and since inducible nitric oxide synthase (iNOS) is activated through the NF-κB pathway [31,32], JasHEx anti-inflammatory activity was evaluated performing a nitric oxide assay. RAW 264.7 cells were treated with the extract (0.0006%, 0.002% and 0.006% p/v) or with the positive control TPCK (10 μM) and then, were stressed with LPS (2 μg/mL). The amount of NO was revealed by adding Griess reagent and the absorbance was measured at 540 nm. As shown in Figure 4D, JasHEx reduced the levels of NO approximately by 30% at all tested concentrations.

## 4. Discussion

Here, an hydroethanolic extract derived from *Jasminum sambac* cell cultures (JasHEx) was studied. Its GNPS-aided mass spectrometry based chemical characterization revealed the presence of phenolic acid derivatives (mainly chlorogenic acids), lignans (secoisolariciresinol, nortrachelogenin and matairesinol) and triterpenes (arjunolic acid, asiatic acid, maslinic acid, oleanolic acid and ursolic acid). All of these secondary metabolites possess well-known antioxidant properties. Indeed, chlorogenic acids [25] and lignans [26,33,34], thanks to their phenolic moiety, have free radical scavenging and chain-breaking antioxidant activities: they donate hydrogen atoms to free radicals, giving rise to phenoxyl radicals stabilized by resonance, and thereby inhibiting the propagation of radical chain reactions and other biological oxidants. Moreover, they act as secondary antioxidants by binding metal ions (Fe(III) and Cu(II)) able to catalyze oxidative processes. Moreover, thanks to an accurate quantitative analysis, it emerged that JasHEx contains relevant amounts of arjunolic acid, asiatic acid, maslinic acid, oleanolic acid and ursolic acid: all these triterpenes can also act as good free radical scavengers, chain-breaking antioxidants or transition metal chelators [35,36,37,38,39].

On the basis of these results, the antioxidant activity of JasHEx was evaluated. It was able to reduce cytosolic ROS production in H_2_O_2-_stressed keratinocytes. Furthermore, since the conversion step of Amadori products into AGEs is dependent on oxidation reactions [9], the antiglycation potential of the extract was tested. It was confirmed by both in vitro and ex vivo assays: JasHEx reduced AGE formation in glyoxal treated HDF and methylglyoxal stressed skin explants, an already used model to highlight the anti-glycation activity of natural substances [28].

In addition to this, JasHEx also showed an extracellular matrix booster effect increasing the production of collagen type I, that is highly sensitive to glycation [10,11,12] and whose levels are significantly reduced by oxidative stress [4].

In vitro assays proved that the antioxidant properties of JasHEx, such as those of chlorogenic acids [40] and triterpenoids [27], are not only related to free radical scavenging and metal chelating activities, but also to the enhancement of Nrf2/ARE pathway. This is the most pivotal endogenous antioxidative system studied so far: when cells are exposed to stressing conditions, Nrf2 dissociates from its cytoplasmic repressor kelch-like ECH-associated protein 1 (Keap1) and translocates to the nucleus where interacts with ARE, activating the transcription of its target genes; the expression of these genes involved in detoxification, NADH regeneration, glutathione (GSH) and thioredoxin (TXN)-based antioxidant system, lipid, eme and iron metabolism increases cell resistance to oxidative stress [41,42].

Moreover, the extract also showed an anti-inflammatory activity, decreasing the release of NO in LPS-stimulated macrophages. This effect is also related to the triggering of Nrf2/ARE pathway: Nrf2 upregulates the expression of HO-1, that, creating a more reducing environment, inhibits the activation of the pro-inflammatory transcription factor NF-κB [31].

## 5. Conclusions

On the basis of the chemical composition and the biological activity proved by in vitro and ex vivo experiments, JasHEx can be considered as a natural powerful antioxidant booster against oxidative stress-induced skin aging.

## Figures and Tables

**Table 1:** Molecular formula (MF), retention time (RT), MS data of compounds identified in JasHEx by GNPS search library and literature study.

	Compound	MF (Mass Error ppm)	RT min	Precursor Ions *m*/*z*	MS^2^ Ions *m*/*z* (Relative Intensity %)
**1**	L-Histidine	C_6_H_9_N_3_O_2_ (2.60 ppm)	1.49	154.0615	137.0346 (37.67); 110.0712 (16.55); 93.0446 (40.25)
**2**	Pyridoxine	C_8_H_11_NO_3_ (2.98 ppm)	1.56	168.0660	150.0551 (100); 122.0600 (46.71)
**3**	Pyridoxal	C_8_H_9_NO_3_ (3.01 ppm)	1.56	166.0504	138.0551 (100); 108.0443 (56.53)
**4**	L-Asparagine	C_4_H_8_N_2_O_3_ (1.53 ppm)	1.64	131.0453	114.0185 (100); 95.0239 (21.51); 70.0286 (38.76)
**5**	L-Glutamine	C_5_H_10_N_2_O_3_ (2.07 ppm)	1.65	145.0611	127.0502 (93.52); 109.0396 (31.30); 84.0442 (21.65)
**6**	L-glutamic acid	C_5_H_9_NO_4_ (1.37 ppm)	1.81	146.0450	128.0342 (52.95); 102.0548 (100)
**7**	Uridine	C_9_H_12_N_2_O_6_ (5.35 ppm)	1.85	243.0625	200.0558 (9.58); 110.0236 (100)
**8**	Quinic acid	C_7_H_12_O_6_ (4.19 ppm)	1.97	191.0558	173.0445 (2.07); 127.0389 (3.40)
**9**	Niacin	C_6_H_5_NO_2_ (0.82 ppm)	2.73	122.0238	94.0286 (5.06); 78.0336 (3.99)
**10**	L-Tyrosine	C_9_H_11_NO_3_ (3.89 ppm)	2.73	180.0662	163.0392 (100); 119.0491 (67.29); 93.0334 (24.76)
**11**	Malic acid	C_4_H_6_O_5_ (2.26 ppm)	2.75	133.0134	115.0025 (100); 71.0126 (45.50)
**12**	Citric acid	C_6_H_8_O_7_ (4.19 ppm)	2.82	191.0194	111.0076 (11.67); 85.0282 (100)
**13**	Guanosine	C_10_H_13_N_5_O_5_ (6.38 ppm)	3.01	282.0851	150.0411 (100); 133.0145 (7.54)
**14**	Adenosine	C_10_H_13_N_5_O_4_ (6.39 ppm)	3.23	266.0901	134.0461 (100)
**15**	L-Phenylalanine	C_9_H_11_NO_2_ (3.05 ppm)	4.34	164.0711	147.0442 (100); 72.0079 (34.30)
**16**	Pantothenic Acid	C_9_H_17_NO_5_ (5.04 ppm)	7.10	218.1034	146.0813 (73.48); 88.0392 (100)
**17**	L-Tryptophan	C_11_H_12_N_2_O_2_ (4.43 ppm)	7.37	203.0824	142.0652 (27.99); 116.0494 (100); 74.0235 (43.29)
**18**	Caffeoylated monosaccharides [22]	C_15_H_18_O_9_ (5.28 ppm)	11.67; 11.91; 12.70; 13.29	341.0885	179.0342; 161.0235; 135.0441
**19**	Coumaroylated disaccharides	C_21_H_28_O_13_ (4.52 ppm)	13.11; 13.54; 13.74; 14.00	487.1468	307.0828; 163.0392; 145.0285
**20**	‡ Secoisolariciresinol	C_20_H_26_O_6_ (6.09 ppm)	16.76	361.1668	346.1422 (37.86); 179.0706 (26.43); 165.0548 (100)
**21**	‡ Nortrachelogenin	C_20_H_22_O_7_ (4.82 ppm)	17.94	373.1300	327.1242 (6.65); 312.1009 (3.98); 147.0442 (10.56)
**22**	‡ Matairesinol	C_20_H_22_O_6_ (5.04 ppm)	19.62	357.1351	342.1109 (8.61); 209.0816 (4.25); 122.0362 (14.23)
**23**	‡ Arjunolic acid/Asiatic acid	C_30_H_48_O_5_ (4.72 ppm)	24.09	487.3441	421.3155 (0.11); 409.3109 (0.44)
**24**	Glycerophosphocoline (18:3)	C_26_H_48_NO_7_P (4.98 ppm)	25.69	562.3167 (M+HCOOH-H)^-^	277.2173 (100); 224.0690 (13.23)
**25**	Glycerophosphoethanolamine (18:2)	C_23_H_44_NO_7_P (4.20 ppm)	26.69; 27.17	476.2792	279.2328; 196.0375
**26**	Glycerophosphocoline (18:2)	C_26_H_50_NO_7_P (4.08 ppm)	26.75; 27.24	564.3319 (M+HCOOH-H)^-^	279.2330; 224.0691
**27**	Glycoglycerolipid (18:3)	C_27_H_46_O_9_ (3.90 ppm)	27.78	513.3078	277.2171 (100); 253.0928 (5.07)
**28**	Glycerophosphoethanolamine (16:0)	C_21_H_44_NO_7_P (3.76 ppm)	27.66; 28.20	452.2789	255.2328; 196.0374
**29**	Glycerophosphocoline (16:0)	C_24_H_50_NO_7_P (3.89 ppm)	27.73; 28.32	540.3317 (M+HCOOH-H)^-^	255.2328; 224.0689
**30**	Glycerophosphoethanolamine (18:1)	C_23_H_46_NO_7_P (4.39 ppm)	28.54; 29.06	478.2949	281.2485; 196.0374
**31**	Hydroxyoctadecadienoic acid	C_18_H_32_O_3_ (5.08 ppm)	29.12	295.2283	277.2173 (100); 195.1384 (26.92); 171.1019 (45.17)
**32**	‡ Maslinic acid	C_30_H_48_O_4_ (4.24 ppm)	29.71	471.3489	423.3274 (0.39)
**33**	Glycerophosphoethanolamine (18:0)	C_23_H_48_NO_7_P (4.16 ppm)	31.44	480.3105	283.2642 (100); 196.0373 (10.91)
**34**	Octadecatrienoic acid	C_18_H_30_O_2_ (5.41 ppm)	34.05	277.2177	259.2071 (1.72)
**35**	‡ Oleanolic acid/ Ursolic acid	C_30_H_48_O_3_ (5.05 ppm)	34.93	455.3543	407.3312 (0.07)

‡ identification and quantification have been performed through the comparison with the related analytical standard.

**Table 2:** Characteristics of the quantitative evaluation of lignanic and triterpenic compounds.

Compound	Range (nM)	Calibration Curve	R^2^	LOD (nM)	LOQ (nM)
Secoisolariciresinol	50–25,000	y = 2.00E + 08 x	0.9951	3	10
Nortrachelogenin	50–25,000	y = 2.00E + 08 x	0.9938	15	50
Matairesinol	50–25,000	y = 3.00E + 08 x	0.9917	3	10
Arjunolic acid	100–25,000	y = 2.00E + 07 x	0.9929	15	50
Asiatic acid	100–25,000	y = 2.00E + 07 x	0.9865	8	25
Maslinic acid	100–25,000	y = 4.00E + 07 x	0.9957	8	25
Oleanolic acid	100–250,000	y = 2.00E + 07 x	0.9953	0.3	1
Ursolic acid	100–250,000	y = 1.00E + 07 x	0.9935	0.3	1

**Table 3:** Lignanic and triterpenic content.

Compound	Amount (µg/g of Extract)	% RSD
Secoisolariciresinol	0.67	0.94
Nortrachelogenin	39.96	2.46
Matairesinol	6.75	0.65
Arjunolic acid	235.80	0.97
Asiatic acid	336.71	2.55
Maslinic acid	144.02	2.82
Oleanolic acid	214.00	3.57
Ursolic acid	542.80	2.17

## Data Availability

Data are contained within the article and Supplementary Materials.

## Supplementary Materials

The following supporting information can be downloaded at: https://www.mdpi.com/article/10.3390/antiox11122409/s1, Table S1: Molecular formula (MF), retention time (RT), MS data of the identified compounds in the Molecular Networks reported in Figure 2.
