# Supplementary material for: Jasminum sambac Cell Extract as Antioxidant Booster against Skin Aging
Source: Antioxidants (Basel). 2022 Dec 6;11(12):2409. doi: 10.3390/antiox11122409 (PMC9774971; doi:10.3390/antiox11122409)
Supplement: Supplementary file 1 [file antioxidants-11-02409-s001.zip › antioxidants-2067736-supplementary.pdf]

Table S1. Molecular formula (MF), Retention time (RT), MS data of the identified compounds in the Molecular Networks reported in Figure 2.

Compound	MF (Mass error ppm)	RT min	Precursor ions m/z	MS^2 ions m/z (Relative intensity %)
**Quinic acid**	$C_7H_{12}O_6$ (4.19 ppm)	1.97; 2.72; 14.39	191.0558	173.0445; 127.0389
**3-caffeoylquinic acid**	$C_{16}H_{18}O_9$ (5.38 ppm)	13.32	353.0886	191.0555 (100); 179.0343 (39.63); 135.0441 (23.81)
**5-caffeoylquinic acid**	$C_{16}H_{18}O_9$ (5.66 ppm)	14.39	353.0887	191.0554 (100); 179.0343 (4.18); 135.0441 (3.34)
**4-caffeoylquinic acid**	$C_{16}H_{18}O_9$ (5.10 ppm)	15.34	353.0885	191.0554 (66.37); 179.0342 (75.16); 173.0447 (100)
**5-feruloylquinic acid**	$C_{17}H_{20}O_9$ (5.45 ppm)	16.34	367.1044	193. 0500 (13.06); 191.0554 (100); 134.0363 (6.75)
**4-coumaroylquinic acid**	$C_{16}H_{18}O_8$ (6.53 ppm)	16.74	337.0940	173.0447 (100); 163.0392 (24.31); 93.0333 (14.61)
**4,5-dicaffeoylquinic acids**	$C_{25}H_{24}O_{12}$ (4.08 ppm)	18.58	515.1205	353.0882 (5.67); 179.0342 (75.69); 173.0447 (100)
**3,4-dicaffeoylquinic acids**	$C_{25}H_{24}O_{12}$ (4.08 ppm)	19.60	515.1205	353.0883 (4.42); 335.0782 (0.51); 179.0342 (74.88); 173.0447 (100)
**3-coumaroyl-4-caffeoylquinic acid**	$C_{25}H_{24}O_{11}$ (4.81 ppm)	19.80	499.1259	353.0887 (1.52); 173.0447 (100); 163.0391 (14.24); 119.0491 (1.49)
**4-caffeoyl-3-feruloylquinic acid**	$C_{26}H_{26}O_{12}$ (3.78 ppm)	20.14	529.1361	353.0882 (2.42); 173.0447 (100); 134.0360 (1.52)

**Caffeoyl quinic acid lactone (or caffeoyl skimic acid) mono-oxidized form**	$C_{16}H_{14}O_8$ (6.31 ppm)	14.84	333.0626	177.0186 (12.08); 133.0284 (100); 93.0334 (4.39)
**Dicaffeoyl quinic acid lactone (or dicaffeoyl skimic acid) mono-oxidized form**	$C_{25}H_{20}O_{11}$ (4.65 ppm)	17.36	495.0945	177.0186 (15.72); 133.0285 (100); 93.0334 (25.26)
**Dicaffeoylquinic acid mono-oxidized form**	$C_{25}H_{22}O_{12}$ (5.65 ppm)	18.67	513.1057	351.0718; 177.0186 (22.11); 173.0447 (100); 133.0285 (42.45)
**Dicaffeoylquinic acid di-oxidized form**	$C_{25}H_{20}O_{12}$ (4.70 ppm)	18.66	511.0895	351.0727; 177.0186 (7.97); 173.0447 (4.58); 133.0285 (100)

**Feruloyl monosaccharide**	$C_{16}H_{20}O_9$ (5.91 ppm)	12.15	355.1045	235.0601; 193.0499 (18.43); 175.0393 (100)
**Feruloylated disaccharides**	$C_{22}H_{30}O_{14}$ (3.87 ppm)	13.62; 13.93; 14.29	517.1572	337.0929; 193.0500; 175.0393
